# Optimizing Nozzle Structure and Parameters for Continuous Fiber Prepreg Filament 3D Printing

**DOI:** 10.3390/polym17081014

**Published:** 2025-04-09

**Authors:** Sheng Qu, Qi Zhang, Beiying Liu, Wei Li, Yesong Wang, Feilong Li, Jisheng Liu

**Affiliations:** 1 School of Mechanical Engineering, University of Science and Technology Beijing, Beijing 100083, China; d202210305@xs.ustb.edu.cn (S.Q.); m202420724@xs.ustb.edu.cn (Q.Z.); liwei@me.ustb.edu.cn (W.L.); liujisheng@ustb.edu.cn (J.L.); 2 School of Mechanical Engineering, Jiangsu University of Science and Technology, Zhenjiang 212000, China; 231210201310@stu.just.edu.cn; 3 School of Aerospace Engineering and Applied Mechanics, Tongji University, Shanghai 200092, China; 4 NCS Testing Technology Co., Ltd., Beijing 100081, China

**Keywords:** continuous fiber prepreg filament, 3D printing, nozzle, process parameters

## Abstract

Successful continuous fiber-reinforced composite filament 3D printing hinges on the synergistic relationship between the printing nozzle and precisely controlled process parameters. This research uses a simulation model to investigate how printing temperature, printing speed, and nozzle length affect the prepreg filament’s molten state during 3D printing. We employed the Box–Behnken response surface methodology to optimize these key parameters. Using continuous fiber-reinforced composite filament polylactic acid (CFRCF/PLA) as an example, and a printing nozzle with a 1 mm diameter and an 8 mm length of heating zone were designed. The optimal printing parameters were determined to be as follows: printing temperature of 220 °C, printing speed of 300 mm/min, and printing layer height of 0.2 mm. Experimental validation using the optimized nozzle and parameters demonstrated enhanced stability in continuous fiber prepreg filament printing.

## 1. Introduction

**Figure 1:**
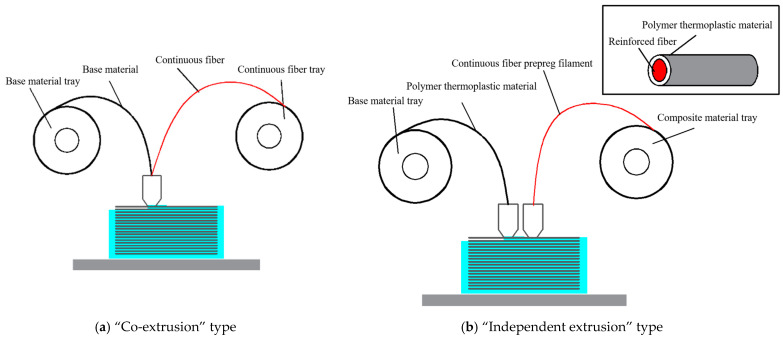
Schematic diagrams of two CFRC 3DP technologies.

Continuous fiber-reinforced composite 3D printing has emerged as a composite fabrication technology developed from Fused Deposition Manufacturing (FDM) [1]. Based on the fiber impregnation methodology, it is categorized into two types: “co-extrusion” type continuous Fiber-reinforced composite 3D Printing (CFRC 3DP) [2,3] and “independent extrusion” type [4], as illustrated in Figure 1. The “co-extrusion” method involves simultaneous feeding of continuous dry fibers and thermoplastic resin matrix into the printing nozzle at a specified ratio during deposition [5,6]. Therefore, this approach suffers from insufficient impregnation time and suboptimal resin-fiber integration, leading to compromised mechanical properties [7]. In contrast, the “independent extrusion” method requires pre-fabrication of continuous fiber prepreg filament [8], offering advantages such as flexible fiber placement, superior resin impregnation efficiency, high printing efficiency, and improved structural integrity. It is a critical pathway for producing lightweight and high-strength composite structures. Nevertheless, the fabrication process for continuous fiber prepreg filament is technologically complex. Due to international technical restrictions, progress in this field has been limited, particularly in achieving high-reliability printing of continuous fiber prepreg filament, which demands urgent exploration and systematic investigation.

The primary technical challenge in continuous fiber prepreg filament 3D printing lies in the design of the printing nozzle, specifically how to cut continuous fiber in the printing nozzle. Zheng et al. [9] designed a continuous fiber-reinforced composite filament (CFRCF) cutting device composed of a motor, leadscrew, and blade to solve the lifting and jumping of the printhead in the process of CFRCF printing. The results show that the device can reliably cut CFRCF and produce a smooth fracture. But the whole printhead is too large and has poor integration. Li et al. [10] designed a printing nozzle for “co-extrusion” CFRC 3DP and further optimized it based on finite element simulation.

The quality of the printed product of continuous fiber prepreg filament is not only reflected on the design of the printing nozzle but also on the printing process parameters. The process parameters include printing temperature, printing speed, wire feed speed, and printing layer height etc., which coupled with each other, directly or indirectly affecting the mechanical properties, surface quality, and production efficiency of the printed product [11,12]. Temperature has a significant impact on the interfacial properties between continuous fibers and matrix materials and is one of the most common parameters influencing the mechanical properties of 3D-printed composite. For example, Tian et al. [13] found that when the printing temperature was lower than 180 °C, the interface performance of continuous carbon fiber-reinforced composite/PLA (CCFRC/PLA) was poor, and the interface was significantly improved when the temperature rose to 240 °C. Printing speed mainly affects forming efficiency. In addition, the printing speed of CFRCF also affects the bonding condition between CFRCF and the printing surface. Faster printing speed may result in insufficient bonding force between CFRCF and the printing surface resin, leading to printing failure, while slower printing speed affects forming and production efficiency. Therefore, it is necessary to achieve a balance between forming efficiency and printing speed. The layer height in the printing process exerts a certain pressure on CFRCF, which can better enhance the interfacial bonding performance [14,15]. However, too small a layer height can cause fiber breakage and nozzle blockage [16]. Blok and Chacóna et al. [17,18] used Markforged printing equipment to research the relationship between different CFRCF (reinforcements: carbon fiber, glass fiber, and Kevlar; matrix material: nylon), different printing parameters (including printing layer height, temperature, and speed, among others), and different mechanical property indicators (tensile/bending strength). Similarly, José Humberto S. et al. [19] used the Anisoprint printing equipment Composer A4 to research the relationship between printing layer height, temperature, speed, and short beam strength of continuous carbon fiber-reinforced PETG composite using statistical method. Li and Bahri Barış Vatandaş et al. [20,21] researched the effect of printing parameters (printing temperature, speed, layer height) in vacuum and atmosphere on the mechanical properties of different CFRCF (reinforcements: carbon fiber, matrix materials: nylon, PEEK) composite samples, and the results showed that the impregnation effect in vacuum was significantly improved compared to that in atmosphere. Through the above investigation, it is found that a large number of experts and scholars generally research the mechanical properties of 3D-printed carbon fiber-reinforced composite structures through printing parameters such as printing temperature, printing speed, layer height, and orientation [22]. In addition to the above research on printing parameters, some scholars have optimized other printing parameters, such as fiber angle, different layer sequences, and filling density [23,24,25,26]. For example, Basim El Essawi et al. [27] found that the position of the continuous carbon fiber layer had a minor effect on the tensile properties of continuous carbon fiber-reinforced nylon composite. From the above research, it was found that there is a certain relationship between the 3D printing nozzle structure and printing process parameters of continuous fiber prepreg filament. Taasnim Ahmed Himika et al. [28] researched the effect of circular and square nozzle shapes and fiber angles on tensile properties. So far, there has been no research on the coupling relationship between 3D printing nozzle structure and printing process parameters.

While existing research has explored individual aspects of nozzle design and process parameter optimization for continuous fiber-reinforced composite 3D printing, a comprehensive understanding of the intrinsic coupling mechanisms between these factors and their impact on printing stability is lacking. This study aims to address this gap by establishing a simulation model to investigate the combined effects of printing temperature, printing speed, and nozzle geometry on the molten state of the prepreg filament and subsequently optimize these parameters for enhanced printing stability.

Taking CFRCF-PLA as an example, this paper researched the relationship between the printing process parameters and the nozzle structure through finite element simulation (Section 2.1). Based on the Box–Behnken response surface method, the key parameters affecting the printing process were decomplicated and optimized. A regression model of the minimum temperature at the CFRCF end face at the nozzle outlet and the length of the heating zone, printing temperature, and printing speed was established (Section 2.2). The optimal combination of CFRCF printing parameters and the nozzle structure parameters was optimized. Finally, through the analysis of the test results, the influence law of printing process parameters on nozzle structure was verified (Section 3.1). CFRCF printing tests were carried out using the optimized printing process parameters and nozzle (Section 3.2). The research of this paper can provide a theoretical basis for the optimization of printing processes and nozzle design of different types of CFRCF.

## 2. Materials and Methods

**Figure 2:**
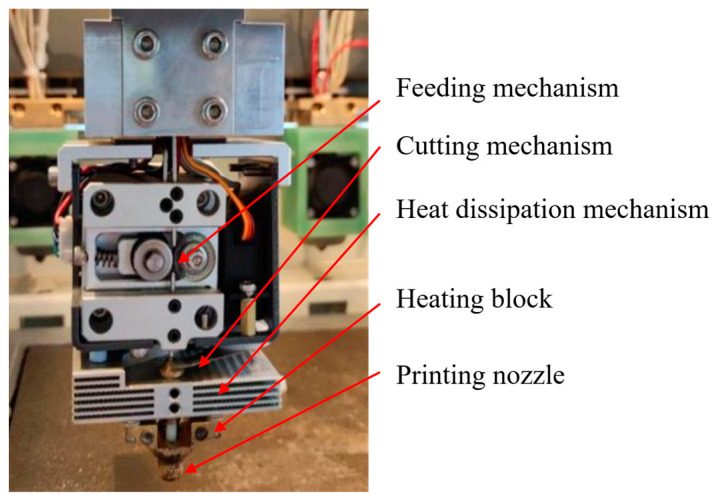
Continuous Fiber Prepreg Filament 3D Printing Nozzle.

The core of a continuous fiber prepreg filament 3D printer lies in the printing nozzle. In this paper, an independently designed CFRCF printing nozzle is used, which has functions such as feeding, heating, heat dissipation, cooling, and cutting, as shown in Figure 2. The feeding mechanism consists of an active gear and a passive rubber wheel, which provides sufficient friction for the feeding of CFRCF. The heating mechanism consists of a heating block and a printing nozzle. The heating block provides heat to the nozzle. When CFRCF passes through the nozzle, it needs to obtain enough heat to melt the resin material wrapped on the surface, ensuring that the melted resin material bonds with the material laid on the upper layer. The heat dissipation and cooling mechanism consists of a heat sink and a cooling fan, which can optimizes reasonably the temperature gradient of the print head, ensuring the smooth printing of CFRCF. The cutting mechanism consists of a servo motor and a blade; when cutting, the servo motor drives the blade to move and cut the CFRCF.

The CFRCF printing nozzle is the core component of the print head, which determines whether the CFRCF printing process can be carried out smoothly. The design of CFRCF nozzle mainly involves two aspects, namely nozzle diameter and the length of the heating zone. Among them, the nozzle diameter is determined by the diameter of CFRCF, the type of surface wrapped resin material, and the printing layer height. The length of the CFRCF 3D printing nozzle (i.e., the length of the nozzle heating zone) is related to the printing temperature, printing speed, and the material wrapped on the outer layer of the composite material wire.

In this paper, self-made continuous fiber PLA pre-impregnated wire (CFRCF/PLA) is used as the test material, and the resin material is PLA produced by eSUN of Shenzhen Guanghua Weiye Co., Ltd. (Shenzhen, China). The detailed parameters are shown in Table 1.

### 2.1. The Relationship Between Printing Process Parameters and Nozzle Structure

As CFRCF passes through the heating zone of the printing nozzle, the nozzle heating zone heats the CFRCF, causing the resin material wrapped on its surface to melt. Subsequently, it bonds and then solidifies with the printing substrate. Due to the influence of printing speed, the residence time of CFRCF in the nozzle heating zone is extremely short. Moreover, CFRCF printing requires the rapid melting of the resin material wrapped on the CFRCF surface. Therefore, using a nozzle with a high temperature and a short heating zone to achieve the rapid melting of the resin material wrapped on the CFRCF surface is key to CFRCF printing. Taking CFRCF/PLA as an example, if the resin material wrapped on the surface of CFRCF is fused and bonded to the printing substrate, the minimum temperature *T_p_* at the end face of the CFRCF at the nozzle outlet must be higher than the minimum printing temperature of the resin. For instance, if the material on the surface of the CFRCF surface is PLA, *T_p_* needs to be higher than the minimum printing temperature of PLA, which is 180 °C. *T_p_* is also related to the length of the heating zone *L* of the CFRCF printing nozzle, the heating temperature *T* of the nozzle heating zone (i.e., the CFRCF printing temperature), the feeding speed *V* of the CFRCF (i.e., the CFRCF printing speed), the nozzle diameter *D*, and the CFRCF diameter *d*. It should satisfy the relationship presented in Equation (1). The *T* provides the initial heat input, while the *L* and *V* determine the time available for heat to transfer to the CFRCF. The diameters (*D* and *d*) influence the surface area available for heat transfer.

(1) $$T_{p}=T_{p}(L,T,V,D,d)$$

The heating time *t* of CFRCF in the nozzle heating zone is related to the length of the heating zone *L* and the printing speed *V*, and it should satisfy the relationship shown in Equation (2). Given a fixed length of *L*, increasing *V* reduces the amount of time the CFRCF spends being heated. This has direct implications for the resin melting process, as insufficient heating time can lead to incomplete melting and poor layer adhesion.

(2) $$t=\frac{L}{V}$$

CFRCF is a self-made material with a fixed diameter *d*. In this case, the maximum diameter type of CFRCF material is selected, with *d* = 0.45 mm. The nozzle diameter *D* of CFRCF is taken as 1 mm. While *D* and *d* will affect the magnitude of heat transfer, they do not fundamentally change the relationship between *L*, *T*, *V*, and *T_p_* within the ranges we studied. Therefore, Equation (1) can be simplified to the form shown in Equation (3).

(3) $$T_{p}=T_{p}(L,T,V)$$

From Equation (3), the minimum temperature of the end face of the CFRCF at the nozzle wire position (*T_p_*), that is, whether the CFRCF can be printed smoothly depends on the nozzle length *L*, printing temperature *T* and printing speed *V*. According to the above analysis, it can be concluded that theoretically, there should exist a length of the heating zone *L* = {*L_a_*, *L_b_*}, printing temperature *T* = {*T_a_*, *T_b_*}, and printing speed *V* = {*V_a_*, *V_b_*} that can achieve the rapid melting of CFRCF and meet the forming requirements of CFRC 3DP. A reasonable length of the heating zone *L* and printing speed *V* correspond to a reasonable heating time interval *t* = {*t_a_*, *t_b_*}, that is, the printing temperature *T* and the time *t* that CFRCF passes through the nozzle heating zone should satisfy the relationship shown in Table 2.

### 2.2. Modeling of the Continuous Fiber Prepreg Filament Printing Process

In the printing process of continuous fiber prepreg filament, it can be regarded as moving downward along the inner wall of the nozzle. As time progresses, the temperature of the continuous fiber prepreg filament gradually increases from low to high, eventually reaching the set temperature of the nozzle. Under different speeds, the length of the nozzle required to reach the set temperature of the nozzle varies. In order to establish a simulation model for the CFRCF printing process, the following assumptions are made considering the main factors and ignoring the secondary factors.

(1) It is assumed that the properties of CFRCF after synthesis are determined by the resin material wrapped on its surface. In the prepared CFRCF, the properties of the continuous fiber pure filament (such as glass fiber, carbon fiber, etc.) are stable, and the preparation method of CFRCF is a physical method, which does not affect the performance of the resin material wrapped on its surface. Therefore, it can be assumed that the performance of CFRCF during the printing process is determined by the properties of the resin material wrapped on its surface.

(2) It is assumed that the shape of CFRCF does not change during the heating process. CFRCF contains a large amount of continuous fiber pure filament, and the resin material wrapped on the surface of CFRCF should originally be in a flowing state after heating. However, due to the restraint of the continuous fiber pure filament, it cannot flow. Therefore, the change in the shape of CFRCF during the heating process is so small that it can be neglected.

(3) It is assumed that the properties of the resin material wrapped on the surface of CFRCF do not change in certain temperature and state conditions. The impregnation material in continuous fiber-reinforced composite will experience slight changes in performance parameters during the heating process, such as density, thermal conductivity, specific heat capacity, etc. Research has found that temperature has little effect on the thermal diffusivity of PLA, and the specific heat capacity value of Polylactic Acid (PLA) changes very little with temperature within a certain range.

(4) It is assumed that the movement of CFRCF in the printing process in the heating zone of the printing nozzle is frictionless and ideal. During printing, CFRCF has certain harden ability, and the diameter of the CFRCF printing nozzle is larger than the diameter of the CFRCF wire. When the CFRCF moves a small distance from the cutting mechanism to the print point at the end of the nozzle, it can be assumed that CFRCF is in a vertical state.

(5) It is assumed that other factors have no effect on the printing of CFRCF.

**Figure 3:**
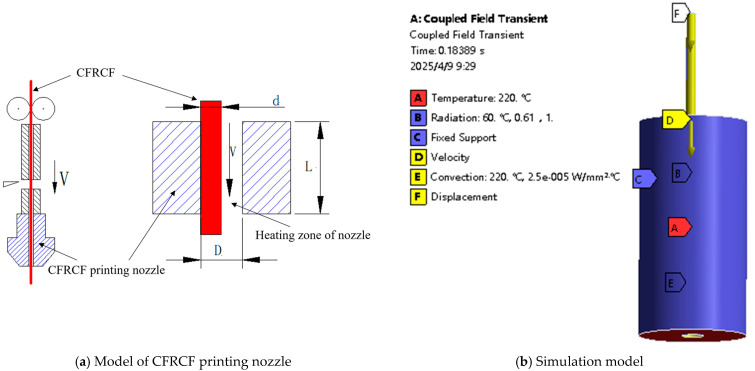
Model of CFRCF printing.

Based on the above assumptions, a simplified model of CFRCF printing is developed, as shown in Figure 3a. Among them, L is the length of the heating zone, D is the nozzle hole diameter, V is the printing speed of CFRCF, and d is the diameter of CFRCF. When CFRCF passes through the nozzle heating zone, it is primarily heated though thermal radiation and conduction. In thermal analysis, the main factors involved are the thermal conductivity, specific heat capacity, radiation coefficient, etc., of the material. Since the calculation model structure is not complicated, the heating block area is finely meshed to ensure high calculation accuracy, as shown in Figure 3b. According to the above analysis, the length of the heating zone of the CFRCF printing nozzle is determined through simulation using the Coupled Field Transient module in ANSYS 2021R2.

The boundary conditions in thermal analysis include temperature, convection, and radiation, among other factors. For the working environment of CFRC 3D printing, the heat source of the nozzle heating zone is selected as stable heat sources of 180, 190, 200, 210, and 220 °C, and the heat conduction occurs at the contact surface between CFRCF and the nozzle heating zone. The material properties are shown in Table 3.

Since brass is easily oxidized to oxidized brass when it is heated in the air for a long time, the radiation coefficient of brass is taken as 0.61, which is the radiation coefficient of oxidized brass, as shown in Table 4. CFRCF is in contact with the nozzle, and heat convection is added. Because the inside of the nozzle is a narrow hole diameter, the environmental temperature is approximately equal to the temperature of the heating zone with air conduction, and heat transfer coefficient is taken as 25 W/m^2^ °C.

### 2.3. Optimization Method for Continuous Fiber Prepreg Filament Printing Process

From the analysis of the printing mechanism of CFRCF, it is known that the length *L* of the heating zone of the printing nozzle, the printing temperature *T*, and the printing speed *V* are intercoupled and jointly determine whether CFRCF can be printed smoothly. The response surface method is employed to decouple and optimize the influencing factors, and the optimal solution is finally calculated according to the actual printing conditions. Taking the length *L* of the heating zone of the CFRCF printing nozzle (Factor 1), the printing temperature *T* (Factor 2), and the printing speed *V* (Factor 3) as parameters, and the minimum temperature of the end face of CFRCF entering the nozzle first (CFRCF minimum temperature) as the response value. The values of each factor are shown in Table 5.

## 3. Results and Discussion

### 3.1. Printing Principle and Scheme Verification Results and Analysis

**Figure 4:**
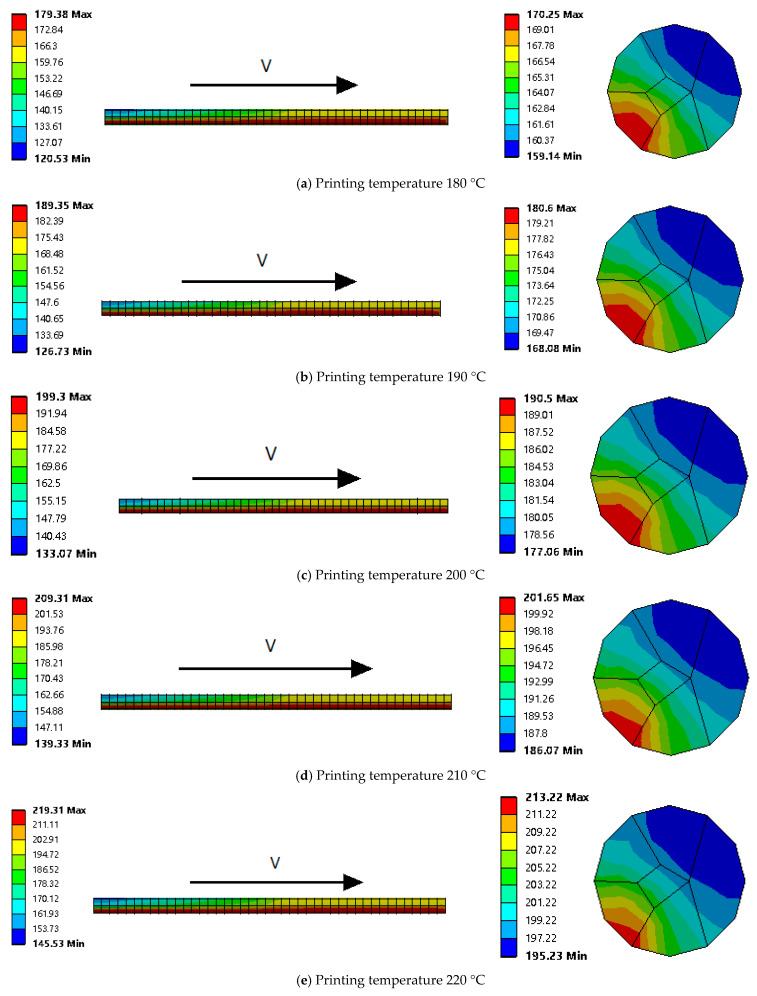
Thermal distribution of CFRCF surface and the end face entering the nozzle first at different printing temperatures.

In order to directly compare the test and simulation results and thus validate the original model, the simulation model was constructed using actual printing parameters, with the CFRCF printing nozzle diameter *D* = 1 mm, nozzle length *L* = 10 mm, CFRCF diameter *d* = 0.45 mm, printing temperature *T* ranging from 180 °C to 220 °C, and printing speed *V* = 300 mm/min [10,29]. Through simulation and actual printing phenomena, it was verified whether the conditions for CFRCF printing were met. The thermal distribution on the surface of the CFRCF and the thermal distribution at the end face of the CFRCF entering the nozzle heating zone first were used for evaluation. The results are shown in Figure 4.

From the figure, it can be seen that as the CFRCF passes through the printing nozzle, the surface thermal distribution indicates that the temperature of the part entering the nozzle first is the highest, and the temperature gradually decreases in the sections further back. Moreover, as the printing temperature increases, the length of the temperature segment on the surface of the CFRCF passing through the printing nozzle that is above the PLA melting point of 160 °C increases because the part entering first is heated for the longest time. In the end face cloud chart, it can be seen that the temperature is highest where the CFRCF contacts the nozzle and diffuses outward from the contact point. When the CFRCF printing temperature is 180 °C, as shown in Figure 4a, the minimum temperature at the end face of the CFRCF at the outlet position after passing through the printing nozzle is only 159.14 °C, which is lower than the PLA melting point of 160 °C. Therefore, when the end face of the CFRCF entering the nozzle first reaches the printing surface, it has not completely melted, and thus, during the printing process, there is a high probability that the CFRCF will fail to print successfully. At the same time, when the CFRCF printing temperature is 190 °C and 200 °C, as shown in Figure 4b,c, the minimum end face temperatures are 168.08 °C and 177.06 °C, respectively. Although the minimum temperature has reached the PLA melting point, there is still a phenomenon of poor adhesion to the printing surface because the recommended minimum printing temperature for the PLA is 180 °C, and the surface viscosity has not met the printing requirements. From Figure 4d,e, it can be seen that when the printing temperature is 210 °C and 220 °C, the minimum temperatures at the end face of the CFRCF entering the nozzle first are 186.07 °C and 195.23 °C, respectively. At this point, the printed CFRCF can reliably bond to the printing surface.

**Figure 5:**
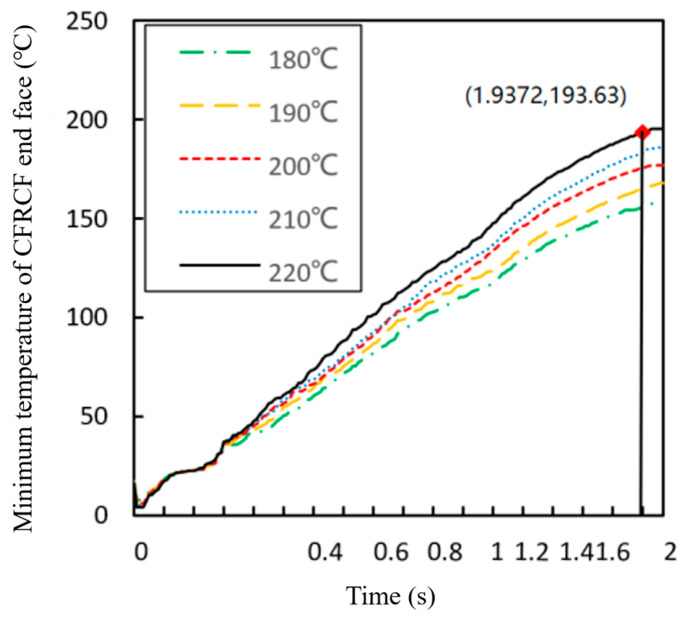
Variation curve of the minimum temperature at the CFRCF end face over time at different temperatures.

**Figure 6:**
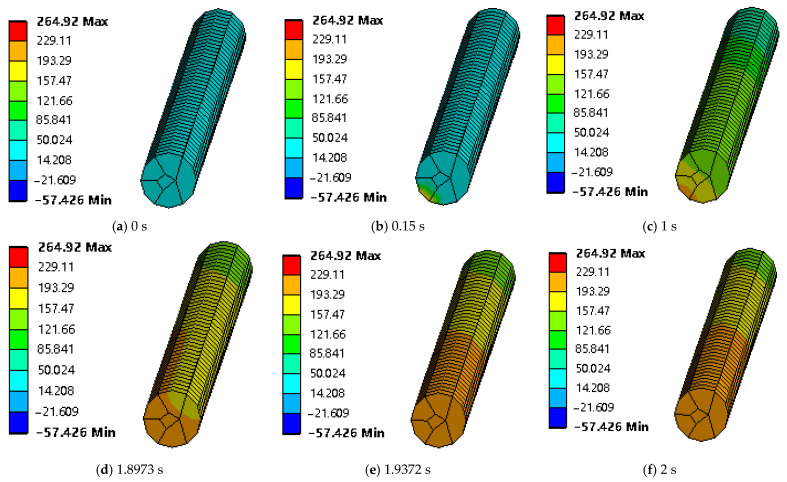
Cloud chart of surface temperature variation in CFRCF at a printing temperature of 220 °C.

Figure 5 shows the variation trend of the minimum temperature at the end face of the CFRCF entering the nozzle first with time in the simulation data. It can be seen from the figure that the minimum temperature value increases with time. When the CFRCF printing temperature is 220 °C and the time for the CFRCF to pass through the nozzle heating zone is 1.9372 s, the minimum end face temperature is 193.63 °C, and then the temperature remains relatively stable. Figure 6 shows the cloud chart of the surface temperature variation in the CFRCF at a printing temperature of 220 °C. Figure 6a shows the surface temperature field is room temperature before CFRCF enters the nozzle. Subsequently, as it enters the nozzle, the heat transfer begins at the contact point of the end face entering first, as shown in Figure 6b. As the time of entering the nozzle increases, the heat transferred from the end face to the upper part of the surface increases progressively. When the entry time is 1.9372 s, as shown in Figure 6e, the temperature of the CFRCF end face reaches 193.29 °C.

### 3.2. Results and Analysis of Continuous Fiber Prepreg Filament Printing Process Optimization

This study utilizes a Box–Behnken experimental design to investigate the influence of three key parameters on the minimum temperature at the CFRCF end face as it enters the nozzle (CFRCF minimum temperature). The three independent variables are as follows: *A*: Length of the heating zone (mm); *B*: Printing temperature (°C); and *C*: Printing speed (mm/min). These parameters were varied across three levels according to the Box–Behnken matrix. The CFRCF minimum temperature (*R*, response value in °C) for each experimental run was obtained using the finite element simulation method. The complete experimental design matrix and the corresponding response values are presented in Table 6.

#### 3.2.1. Model Verification

Analysis of Variance (ANOVA) is used to evaluate the effectiveness of the model in predicting the response value of the studied variable within a certain range and provide a basis for subsequent optimization. Coding factor is used for analysis. Based on sum of squares for Type III, the partial derivative analysis results are shown in Table 7. The F-value of the model in the table is 68.59, and the *p*-value is much less than 0.0001, indicating that the model has significance of difference. The R^2^ value was 0.9888, suggesting that the regression equation accurately fits the experimental data, explaining 98.88% of the variance. However, the predicted R^2^ value of 0.8205, in contrast to the high R^2^, raises concerns about potential overfitting, suggesting that the model’s predictive accuracy may be less reliable outside the experimental design space defined by the Box–Behnken matrix, as shown in Table 6. The *p*-values of *A*, *B*, *C*, *AC*, *A*^2^, *B*^2^, and *C*^2^ are all less than 0.05, indicating that they have significant effects on the model. According to the F-value test, the order of importance of the factors affecting the minimum temperature at the end face of CFRCF is printing speed, length of the heating zone, and printing temperature, respectively. The interaction effects of the three factors on the minimum temperature at the end face of CFRCF are in the order of *AC* > *BC* > *AB*, the practical implications of which are discussed in Section 3.2.2. The Adeq precision is used to measure the signal-to-noise ratio, and its value is 32.222, which is much greater than the ideal value of 4, indicating that the established model can be used for response surface optimization.

The regression equation between the length of the heating zone (*A*) of the nozzle, the printing temperature (*B*), the printing speed (*C*), and the minimum temperature (*R*) at the end face of CFRCF entering the nozzle first, obtained by fitting the experimental results, is shown in Equation (4).

(4) $$R=142.38+27.46A+13.41B-42.56C+2.98AB+17.16AC-4.46BC-17.04A^{2}-9.74B^{2}+26.37C^{2}$$

#### 3.2.2. Parameter Optimization

**Figure 7:**
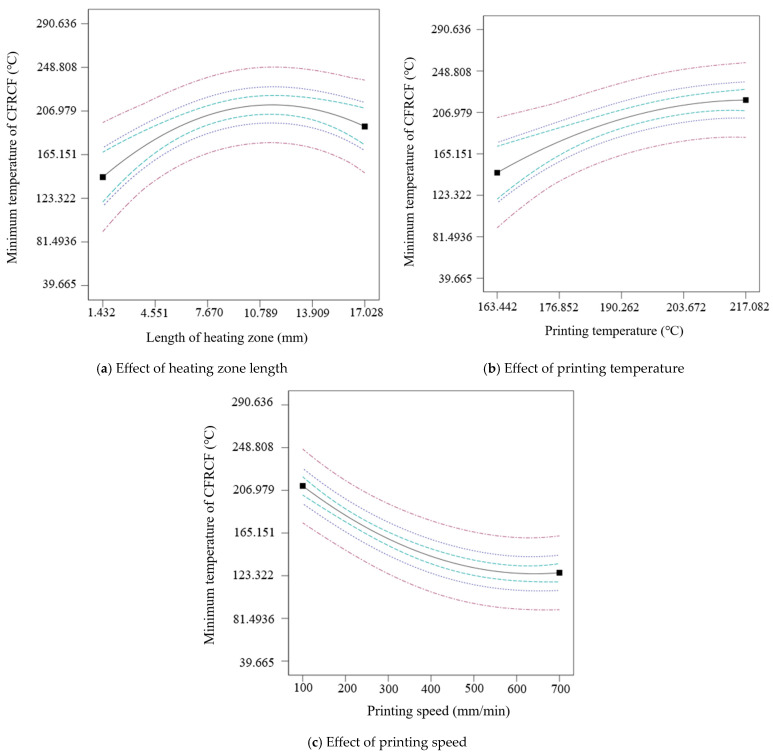
Curves of the effects of single factors on the minimum temperature of CFRCF.

Figure 7 shows the curves of the effects of single factors on the minimum temperature at the end face of CFRCF entering the nozzle first. The minimum temperature at the end face of CFRCF increases with the increase in the length of the heating zone and printing temperature, as shown in Figure 7a,b, and decreases with the increase in printing speed, as shown in Figure 7c. The higher the printing temperature, the more energy is available for heating the surface of CFRCF. A longer heating zone and slower printing speed indicate a longer heating time for CFRCF, resulting in more heat energy obtained by CFRCF as it passes through the heating zone. Moreover, from Figure 7a, it can be seen that when the length of the heating zone exceeds a certain length, the change in the minimum temperature of CFRCF will tend to slow down. When the length of the heating zone is already large enough to maximize the surface temperature of CFRCF, increasing the length of the heating zone will not increase the surface temperature of the CFRCF. From Figure 7c, it can be seen that when the printing speed increases to a certain extent, the change in the minimum temperature of CFRCF becomes slow. It is because when passing through the heating zone, the CFRCF can obtain enough energy to heat its surface to a certain temperature in a short time.

**Figure 8:**
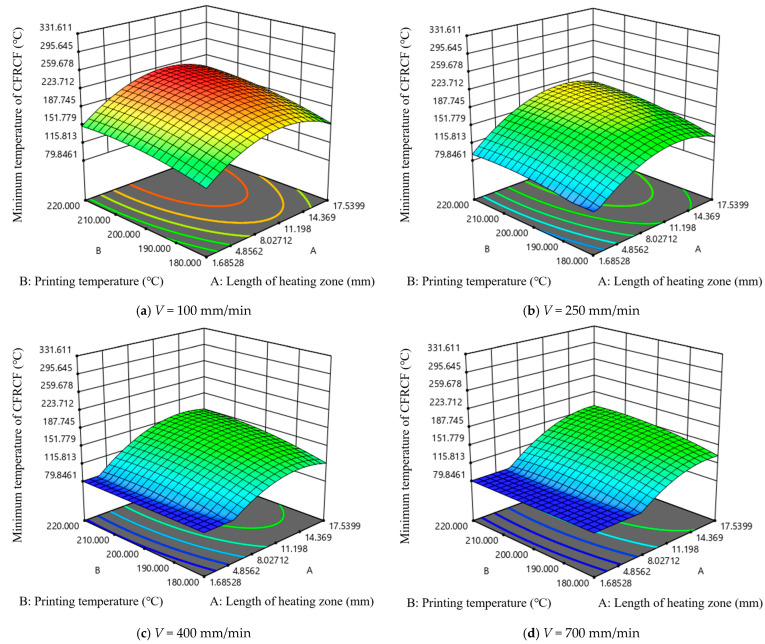
Interactive effect of length of the heating zone and printing temperature on the minimum temperature at the end face of CFRCF.

Figure 8 shows the effect of length of the heating zone and the printing temperature on the minimum temperature at the end face of CFRCF. As the printing speed value increases, the minimum temperature of CFRCF decreases. Under the same length of the heating zone, the trend of the impact of printing temperature on the minimum temperature of CFRCF is very slow in almost all printing speeds. It can be seen from Figure 8b that when the printing speed starts from 250 mm/min, with the increase in printing speed, the length of the heating zone with a slow impact on the minimum temperature of CFRCF gradually increases. It can be clearly seen from Figure 8a that when the printing speed is the lowest and the printing temperature is 220 °C, the minimum temperature value of CFRCF is the highest. This interaction suggests that the effect of nozzle length on printing performance is less dependent on the printing temperature. This might be because, even at lower temperatures, a longer nozzle still provides some benefit in terms of improved filament homogenization, although the effect is less pronounced than at higher temperatures or speeds.

**Figure 9:**
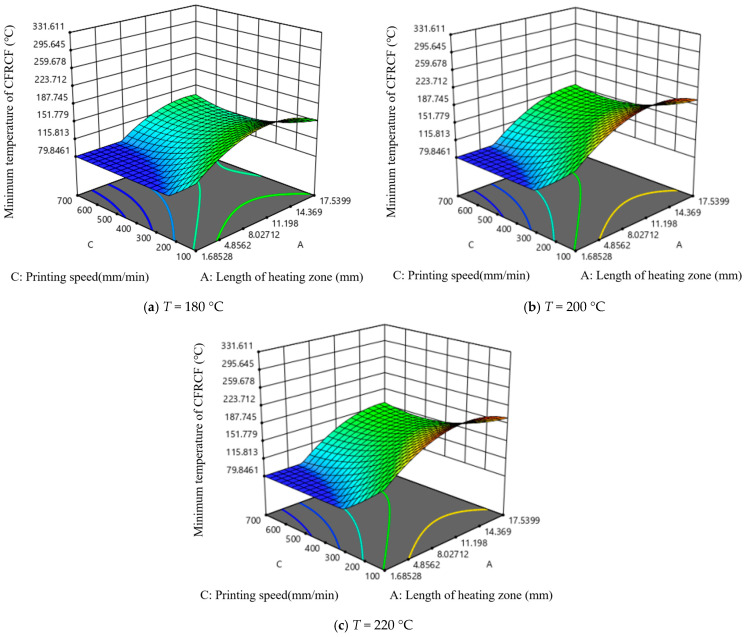
Interactive effect of length of the heating zone and printing speed on the minimum temperature at the end face of CFRCF.

Figure 9 is the interactive effect diagram of length of the heating zone and printing speed on the minimum temperature at the end face of CFRCF. It can be seen that under different printing temperatures, the trend of the impact of length of the heating zone and printing speed on the minimum temperature of CFRCF is basically unchanged. The larger the length of the heating zone and the slower the printing speed, the higher the minimum temperature value of CFRCF. In the region of the small heating zone and fast printing speed, the printing temperature has almost no impact on the minimum temperature of CFRCF. This strong positive interaction suggests that a longer nozzle length is more beneficial at higher printing speeds. This is likely because the longer nozzle provides a longer residence time for the filament to melt and homogenize at the higher throughput rates associated with faster printing speeds. Without sufficient residence time, the filament may not fully melt, leading to poor layer adhesion and printing defects.

**Figure 10:**
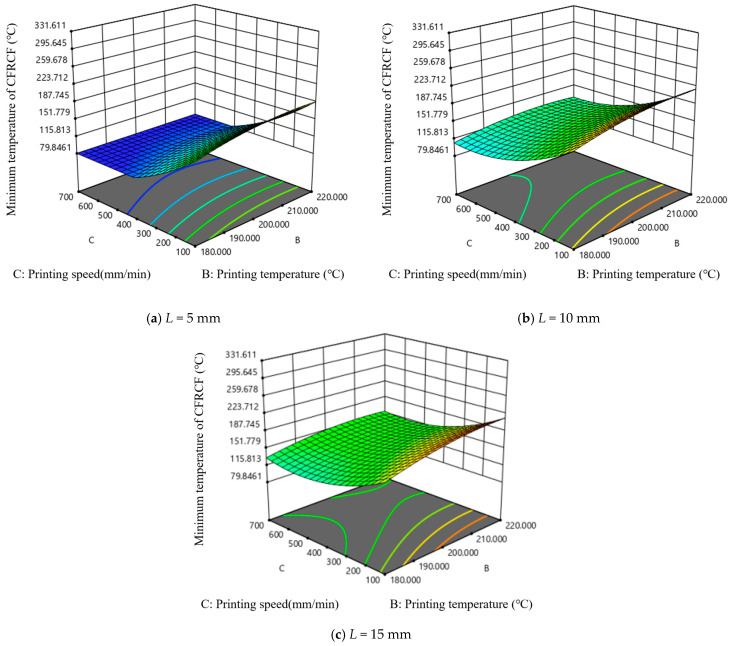
Interactive effect of printing temperature and printing speed on the minimum temperature at the end face of CFRCF.

Figure 10 is the interactive effect diagram of printing temperature and printing speed on the minimum temperature at the end face of CFRCF. It can be seen from the figure that in almost all lengths of the heating zones, in the region where the printing speed is greater than 500 mm/min, the change in printing temperature has a slow impact on the minimum temperature of CFRCF. This interaction suggests that a higher printing temperature is necessary at higher printing speeds. This is because higher printing speeds require more energy input to melt the filament in a shorter amount of time. If the temperature is not sufficiently high, the filament may not reach its optimal viscosity for proper extrusion and layer bonding.

The above simulation analysis is consistent with the actual printing phenomena of CFRCF. When the printing temperature *T*, printing speed *V*, and the length of the heating zone *L* satisfy the relationship in Table 2, the minimum temperature at the end face of CFRCF (*T_p_*) can meet the printing requirements. These interaction effects highlight the importance of optimizing all three parameters (nozzle length, printing temperature, and printing speed) simultaneously to achieve optimal printing performance. For example, if a user wants to increase the printing speed, they must also consider increasing the nozzle length and/or the printing temperature to compensate for the reduced residence time and increased energy requirements. This information can be used to develop optimized printing profiles for different CFRCF/PLA filament formulations and printing applications. Therefore, this model can be used to solve for the optimal parameters.

The length of the heating zone, which is the length of the printing nozzle, is preferably between 5 mm and 15 mm in actual use and tends towards the minimum value. The printing temperature mainly needs to meet the printing conditions of the resin on the surface of CFRCF and is expected to have the same printing temperature range as the matrix material printing head, within the range of 200 °C to 220 °C. The printing speed affects printing efficiency, so it is better to be as high as possible while meeting the printing requirements of CFRCF. The minimum temperature at the end face of CFRCF needs to meet the printing temperature requirement of the surface resin, which is between 180 °C and 210 °C. In addition, to ensure stable printing, it should be close to minimum temperature to avoid rapid melting and nozzle clogging.

**Figure 11:**
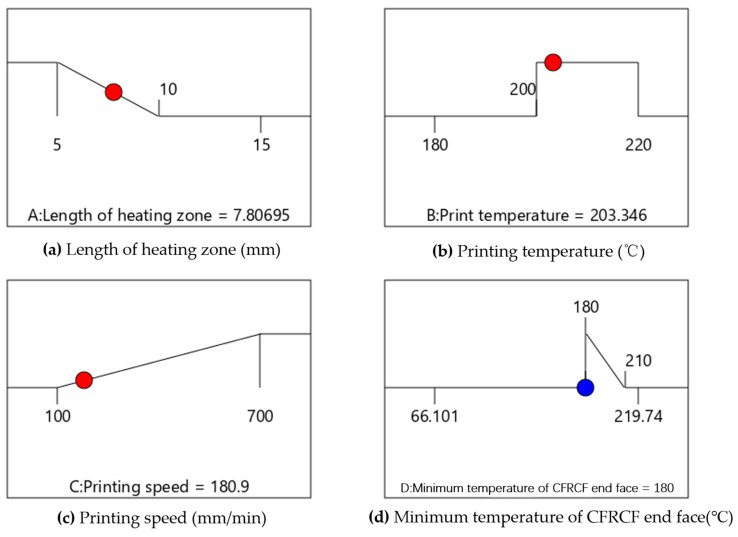
Optimal parameters under suggested process conditions.

The optimal calculation results, obtained by the Box–Behnken response surface method, are shown in Figure 11. It indicates that when the length of the heating zone of the CFRCF printing nozzle *L* equals 7.807 mm, printing temperature *T* equals 203.346 °C, and printing speed *V* equals 180.9 mm/min, the minimum temperature at the end face of CFRCF *T_p_* equals 180 °C, which meets the printing requirements.

**Figure 12:**
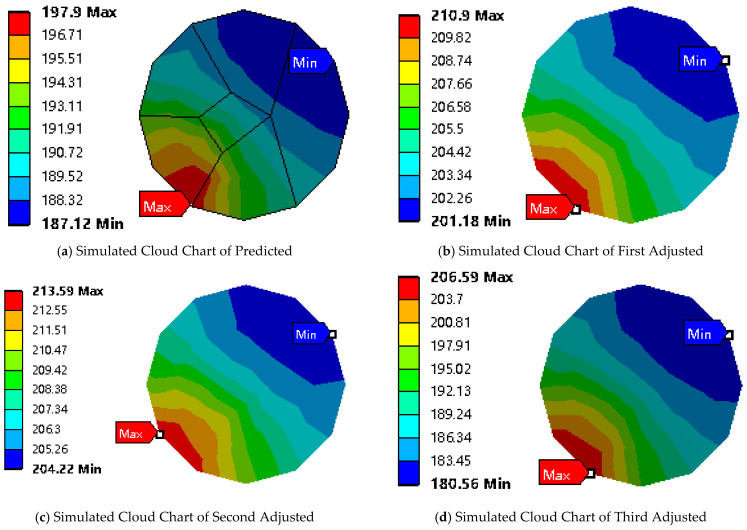
Simulated cloud charts of predicted and adjusted parameters.

The simulation verification of the preferred results is shown in Table 8 and Figure 12a. The minimum temperature at the end face of CFRCF simulated with the predicted parameters is 187.12 °C, which can confirm the reliability of the predictive model.

**Figure 13:**
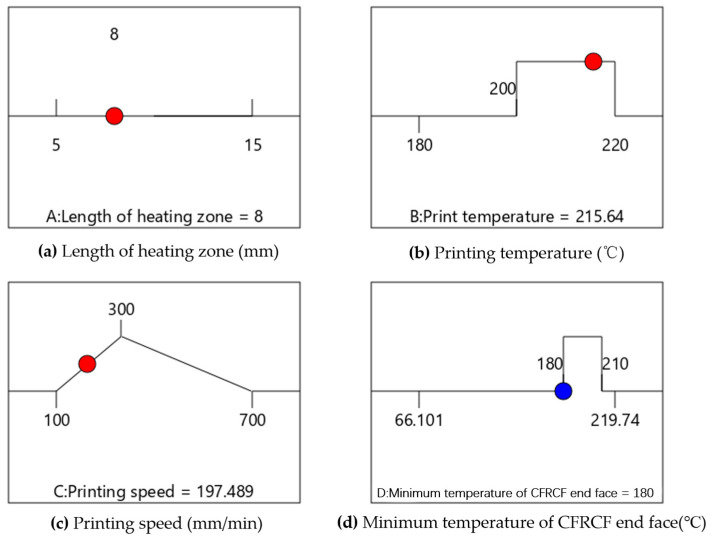
Optimal parameters under the suggested process with *L* = 8 mm.

Because precisely machining the nozzle length to 7.807 mm is difficult, we initially changed it to an easier-to-achieve 8 mm, the optimal parameters calculated by the model are shown in Figure 13. Subsequent simulations with an 8 mm nozzle length resulted in a *T_p_* of 201.18 °C when the printing temperature was 215.635 °C and speed was 197.489 mm/min (First Adjusted Simulated Value in Table 8). The second adjustment considered the recommended printing temperature range (190–220 °C) for the CFRCF/PLA surface resin coating. Aiming for better speed control within this temperature range, we set the printing speed to 200 mm/min. The corresponding simulated *T_p_* was 204.220 °C when the printing temperature was 220 °C and speed was 200 mm/min (Second Adjusted Simulated Value in Table 8). The final adjustment involved increasing the printing speed to 300 mm/min, considering both model predictions and practical printing tests. This final adjustment, influenced by the *BC* interaction in the Box–Behnken design, aimed to improve printing efficiency while maintaining an acceptable surface temperature. The final calculated *T_p_* was 180.56 °C, as shown in Figure 12b, which meets the requirements (Third Adjusted Simulated Value in Table 8).

It is important to discover the initial discrepancy between the predicted and simulated temperatures (180 °C to 187.12 °C for the initial parameters). This difference may arise from model simplifications, such as assuming uniform heat transfer coefficients and ideal material properties, and highlights the inherent limitations of the simulation model in capturing the full complexity of the 3D printing process. However, the simulated value still falls within an acceptable range, supporting the model’s reliability for guiding parameter selection. The data in Table 8 provide insights into the sensitivity of *T_p_* to changes in the parameters. While a formal sensitivity analysis was not conducted, these simulations also show that the printing setting can be improved. The results reveal that the composite material surface temperature is most sensitive to changes in the printing temperature. The other parameters also influence the temperature, but the printing temperature has the most significant impact. This finding supports the strategy of prioritizing printing temperature adjustments in conjunction with printing speed to achieve the desired surface temperature.

In summary, when the CFRCF diameter *d* equals 0.45 mm, the final design of the printing nozzle diameter *D* equals 1 mm, and the length of the heating zone *L* equals 8 mm. The optimal printing parameters for CFRCF/PLA are as follows: printing temperature *T* = 220 °C, printing speed *V* = 300 mm/min, and printing layer height *H* = 0.2 mm.

### 3.3. Experimental Verification

**Figure 14:**
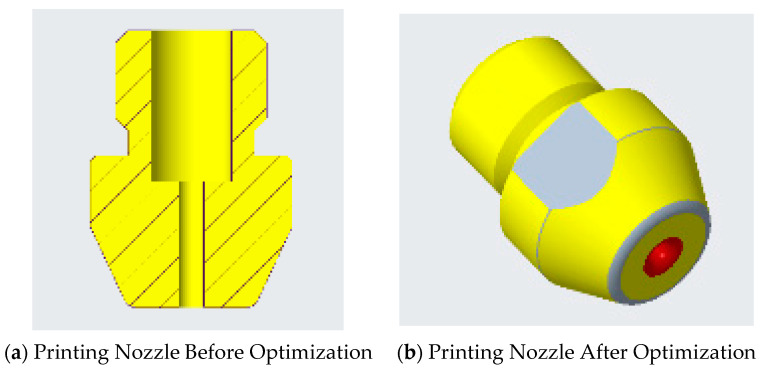
CFRCF printing nozzle before and after optimization.

The optimized printing nozzle was installed on the self-made CFRC 3DP equipment for printing test. Figure 14a shows the CFRCF printing nozzle before optimization. Figure 14b shows the CFRCF printing nozzle after optimization. The detailed parameters of the materials used in the experiment are shown in Table 9.

**Figure 15:**
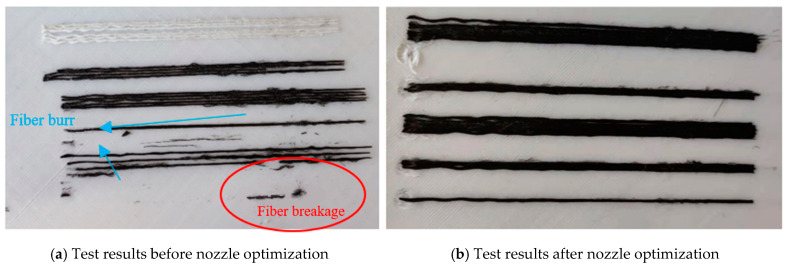
Printing test phenomena.

Figure 15 shows the actual printing test results, where the printing substrate is PLA material, the black part is continuous carbon fiber-reinforced composite/PLA (CCFPF/PLA), and the white part is continuous glass fiber-reinforced composite/PLA (CGFPF/PLA). Figure 15a shows the printing result before nozzle optimization. Initially, we faced challenges with CCFPF/PLA, including fiber breakage and nozzle clogging. The CFRCF would rub against the nozzle outlet edge, causing partial fiber breakage (the burr phenomena shown). Increased breakage led to fiber clogging, resulting in the fiber breakage phenomenon. This underscored the need to optimize the CFRCF printing nozzle. The optimized nozzle structure is shown in Figure 14b, incorporating a transition filet at the nozzle outlet to prevent fiber breakage. Figure 15b shows the printing status after optimization. From the experimental phenomena, it can be seen that the CFRCF can reliably print on the surface. There is no nozzle clogging, and there is a significant improvement in the fiber surface burr phenomena, which verifies that the optimized CFRCF printing nozzle meets the printing requirements.

## 4. Conclusions

Aiming at the phenomenon of the continuous fiber prepreg filament printing nozzle that cannot feed the filament normally and causes nozzle clogging, the internal laws between nozzle diameter, CFRCF diameter, nozzle length of the heating zone, printing temperature, printing speed, and resin material properties were researched. The results show that, theoretically, there should exist a length of heating zone *L* = {*L_a_*, *L_b_*}, printing temperature *T* = {*T_a_*, *T_b_*}, and printing speed *V* = {*V_a_*, *V_b_*} that can achieve the rapid melting of CFRCF and meet the forming requirements of CFRC 3DP. Based on the printing mechanism of CFRCF, the Box–Behnken response surface method was used to decouple and optimize the key parameters affecting the printing process and further optimize the nozzle structure on this basis. Taking CFRCF/PLA as an example, a CFRCF printing nozzle with a nozzle diameter *D* = 1 mm and a heating zone length of *L* = 8 mm was designed. At this point, the optimal printing parameters for CFRCF are as follows: printing temperature *T* = 220 °C, printing speed *V* = 300 mm/min, and printing layer height *H* = 0.2 mm.

This optimized nozzle and parameter set provides a practical solution for reliable CFRCF/PLA printing, addressing common issues such as nozzle clogging and fiber breakage. This enables the production of high-strength, lightweight composite parts with improved efficiency and reduced material waste, which is important for real-world application. Furthermore, the optimization methodology presented in this study can be applied to nozzle design and parameter optimization for a broader range of CFRCF materials, enabling wider adoption of CFRC 3DP technology.

## Figures and Tables

**Table 1:** Detailed Parameters of Test Consumables.

Title	Reinforcement Material	Diameter (mm)	Wrapped Resin	Printing Temperature (°C)	Printing Layer Height (mm)	Source
PLA	-	1.75	-	215	0.2	eSUN
CFRCF/PLA	glass fiber, carbon fiber	0.45	PLA	220	0.2	self-made

**Table 2:** Relationship between Printing Temperature *T*, Heating Time *t*, and CFRC 3DP Forming.

Printing Temperature	Heating Time	Printing Forming Impact
*T > T_b_*	*t > t_b_*	High printing temperature and long heating time result in rapid softening and nozzle clogging of CFRCF.
*T > T_b_*	*t < t_a_*	High printing temperature and short heating time result in ineffective bonding between CFRCF and the printing surface.
*T < T_a_*	*t > t_b_*	Low printing temperature and long heating time result in softening and nozzle clogging of CFRCF.
*T < T_a_*	*t < t_a_*	Low printing temperature and short heating time result in ineffective bonding between CFRCF and the printing surface.

**Table 3:** Material Property Attributes.

Name	Material	Density (kg/m^3^)	Elastic Modulus (GPa)	Poisson’s Ratio	Specific Heat Capacity (J/kg·°C)
Nozzle	Copper alloy	8500	96	0.34	390
CFRCF	PLA	1260	3.45	0.08	1190

**Table 4:** Radiation Coefficient of Brass.

Material	Surface Condition	Radiation Coefficient
Brass	Polished	0.03
	Oxidized	0.61

**Table 5:** Coded Factor Level Parameters.

Factor	Parameter	Level		
		−1	0	1
*A*	Length of the heating zone (mm)	5	10	15
*B*	Printing temperature (°C)	180	200	220
*C*	Printing speed (mm/min)	100	400	700
CFRCF	PLA	1260	3.5	0.08

**Table 6:** Experimental Scheme and Results.

Std	Run	Factor 1	Factor 2	Factor 3	Response 1
		*A*	*B*	*C*	*R*
2	1	15	200	100	199.57
10	2	10	200	400	159.5
15	3	10	200	400	159.5
6	4	5	220	400	116.1
4	5	10	220	100	219.74
14	6	15	220	400	175.87
17	7	5	200	700	66.1
13	8	5	200	100	192.71
3	9	5	180	400	95.52
9	10	10	220	700	128.36
7	11	10	200	400	159.5
16	12	10	180	700	108.29
8	13	15	200	700	148.46
1	14	15	180	400	143.37
5	15	10	180	100	179.67
12	16	10	200	400	159.5
11	17	10	200	400	159.5

**Table 7:** Variance Analysis Results.

Effect	Sum of Squares	df	Mean Square	F-Value	*p*-Value
Model	24,254.62	9	2694.96	68.59	<0.0001
*A*—Length of the heating zone	5732.38	1	5732.38	145.89	<0.0001
*B*—Printing temperature	1366.54	1	1366.54	34.78	0.0006
*C*—Printing speed	14,490.74	1	14,490.74	368.79	<0.0001
*AB*	35.53	1	35.53	0.9043	0.3733
*AC*	1242.58	1	1242.58	31.62	0.0008
*BC*	83.91	1	83.91	2.14	0.1873
*A* ^2^	1223.24	1	1223.24	31.13	0.0008
*B* ^2^	399.43	1	399.43	10.17	0.0153
*C* ^2^	2185.87	1	2185.87	55.63	0.0001
Residual	275.05	7	39.29	-	-
Standard deviation	6.27	-	-	-	-
Median	151.25	-	-	-	-
R^2^	0.9888	-	-	-	-
Adjusted R^2^	0.9744	-	-	-	-
Predictive R^2^	0.8205	-	-	-	-
Adeq Precision	32.2219	-	-	-	-
C.V. %	4.14	-	-	-	-

**Table 8:** Verification Analysis of Box–Behnken Response Surface Method Optimization Results.

	Nozzle Length *L* (mm)	Printing Temperature *T* (°C)	Printing Speed *V* (mm/min)	Composite Material Surface Temperature *T_p_* (°C)
Predicted Value	7.807	203.346	180.900	180.000
Simulated Value	7.807	203.346	180.900	187.120
First Adjusted Predicted Value	8.000	215.635	197.489	180.000
First Adjusted Simulated Value	8.000	215.635	197.489	201.180
Second Adjusted Simulated Value	8.000	220.000	200.000	204.220
Third Adjusted Simulated Value	8.000	220.000	300.000	180.560

**Table 9:** Detailed parameters of the materials used in the experiment.

Title	Reinforcing Material	Diameter (mm)	Wrapped Resin	Printing Temperature (°C)	Printing Layer Height (mm)	Source
printing surface PLA	-	1.75	-	215	0.2	Yisheng Technology
CGFPF/PLA	glass fiber	0.45	PLA	220	0.2	self-made
CCFPF/PLA	carbon fiber	0.4	PLA	220	0.2	self-made

## Data Availability

All relevant data are within the paper.
